# Evansville Medical College

**Published:** 1851-02

**Authors:** 


*Evansville Medical College.*—The Western Medical Chirurgical Journal states that the above named Institution has "generously proposed to admit Sons of Temperance at half their usual fees," whereupon the Sons reciprocate the compliment by publishing that "they have no hesitation in recommending the school as in every way worthy of confidence." The Editor of the Western Medico-Chirurgical, a professor in a neighboring school at Keokuk, Iowa, expresses his scorn of such methods of competition, and adds, "rather than lecture to a class obtained by such means, however large it might be, we would lay aside our professorial robes, and, if need be, take up the language of Francis 1st, " all is lost but our honor!"

